# The Dentist and the Laboratory

**Published:** 1922-02

**Authors:** C. N. Reese

**Affiliations:** Chicago, Ill.


### THE DENTIST AND THE LABORATORY

BY DR. C. N. REESE, CHICAGO, ILL.

In the December issue of *The Dental Digest* the writer gave some general directions which the dental profession should follow in preparing crown, bridge and denture cases for a laboratory, if perfect results are to be expected. The present article seeks to present some supplementary information regarding the technic to be followed in preparing denture cases.

*For perfectly fitting dentures:* Instruct your laboratory to send all full upper and lower plates to you for "try-in" on hard base plates. Moisten the base plate and try the case in the mouth. If it does not stay up, sprinkle a little powdered gum tragacanth over the moistened surface. Pull out the cheeks, note points of muscle interference and trim margins of base plate to permit free play of muscles. Instruct patient to bite; arrange teeth to occlude perfectly, both in the up and down motion and the lateral motion used in chewing. Remove plate carefully from mouth, dry thoroughly and attach all teeth securely with hard sticky wax. Scratch inside of base plate with knife.

Now fill base plate with thin plaster; pour out all the plaster you can; replace a small amount of plaster in center of palate. Replace case in mouth and press up to place, thus taking new impression. Instruct patient to bite lightly and keep the mouth closed until plaster has set. Before taking impression swab the mouth with a mixture of olive oil and glycerine to prevent adhesion of plaster to the tissues.

When the plaster has set remove case carefully from mouth and send to your laboratory for finishing, instructing it to run model in artificial stone.

The same method may be applied to plates which have been worn for a time and have become loose, but are otherwise satisfactory. After taking impression in old plate as above, send to your laboratory and instruct it to reline or duplicate it.

*Full lower dentures:* There are very few lower mouths which can not be fitted with perfectly satisfactory plates if the impression is taken properly. By passing the finger along the lingual surface of the ridge back opposite the base of the tongue, deep undercuts will be found. In most mouths these undercuts are quite deep without any muscle attachments to interfere with edge of plate. An impression should be taken in modelling compound, pressing the compound well down at these points so as to get a deep impression of the undercuts. The inside of the impression should then be trimmed away sufficiently to allow for a fairly thick layer of plaster and a plaster impression taken, using the prepared compound impression as a tray.

*Solarization:* Pink rubber may be bleached to a lighter shade by solarization. Place the finished plate in a glass of alcohol and allow it to stand in the sun for a time. The longer the exposure to the sun the lighter the shade.

*Taking the bite for crown and bridgework:* In some mouths where crowns or bridges are to be made the opposing teeth come into such close contact that the wax or compound is bitten clear through in taking the bite. In such cases, place a layer of wax or compound on each side of a piece of cheese cloth and then take the bite. Try it.

Consult your laboratory regarding your difficult and puzzling cases. The occasion is bound to arise, now and then, in the practice of every dentist where a prosthetic case presents an unusual and difficult problem. To experiment and gradually work out a solution is apt to lose you the confidence of the patient, whereas a quick solution gains his confidence and enhances your professional reputation.

On occasions like this feel free to consult your laboratory man. His experience embraces cases of every degree of difficulty and it is more than likely he can suggest a means of overcoming a mechanical difficulty based on similar cases he has handled in the past.

There is a vast fund of experience stored up in the personnel of a large, well-managed dental laboratory, all of which is at the disposal of the profession.

Above all, be as careful and painstaking with your part of the work as you expect your laboratory man to be with his.

The writer wishes to take this opportunity to warn the profession against price cutting. It is impossible for anyone to cut prices on laboratory work at this time without cutting the quality of materials, substituting cheaper teeth for those ordered or cheapening the workmanship.

There has been no reduction in the cost of high-grade materials or skilled labor, and the prices charged by the established, dependable, ethical laboratories are as low as they can be made. The better laboratories will not cheapen their work by using an inferior quality of materials or slighting their workmanship.

At the same time, appreciating that to the profession "a dollar saved is a dollar earned," some of the larger laboratories are presenting a plan, similar to that offered by most of the progressive dental depots, whereby the profession may save money on their laboratory work without accepting a lower standard of work, by offering a liberal discount for cash in advance. This reduces the cost of financing and enables the laboratory to anticipate material requirements, thus saving money for the dentist without cutting prices or sacrificing quality.

Appliances which are to be inserted into the mouths of human beings *can not be too good,* and the better laboratories will not produce inferior work at lower prices to stimulate trade.

Your patients are entitled to the best. Your reputation depends more upon the quality of your prosthetic work than upon any other class of work you do.

There is a whole lot of pride, good and otherwise, in this world, and many patients will recommend their dentist when his work is what they know their friends would expect.

The dental profession should be, and we are sure is, aware that true quality in laboratory work is obtainable only by a combination of high-grade materials and skilled workmanship. Vital as is the material in this combination its cost is comparatively small compared to the cost of the skilled labor which fashions the inert material into an almost living thing. That is why even reductions in quality materials, when they do come, will but slightly affect the cost of work from those laboratories which have built up and take pride in maintaining a force of highly skilled workmen, real artists in their field.

